# Emotional Modulation of Frontal Alpha Asymmetry - a Novel Biomarker of Mild Traumatic Brain Injury

**DOI:** 10.3389/fnhum.2021.699947

**Published:** 2021-07-20

**Authors:** Venla Kuusinen, Jari Peräkylä, Lihua Sun, Keith H. Ogawa, Kaisa M. Hartikainen

**Affiliations:** ^1^Behavioral Neurology Research Unit, Tampere University Hospital, Tampere, Finland; ^2^Faculty of Medicine and Health Technology, Tampere University, Tampere, Finland; ^3^Turku PET Centre, University of Turku, Turku, Finland; ^4^Department of Psychology, Saint Mary’s College of California, Moraga, CA, United States

**Keywords:** mild traumatic brain injury (MTBI), frontal alpha asymmetry (FAA), emotion, threat, EEG, depression, post-concussion symptoms, biomarker

## Abstract

Objective findings of brain injury or dysfunction are typically lacking in mild traumatic brain injury (MTBI) despite prolonged post-concussion symptoms in some patients. Thus, there is a need for objective biomarkers of MTBI that reflect altered brain physiology underlying subjective symptoms. We have previously reported increased attention to threat-related stimuli in subjects with MTBI, suggesting a physiological vulnerability to depression. Vulnerability to depression has been linked with relatively greater activity of the right than left frontal cortex reflected in inverse pattern in frontal alpha with greater power on the left than right. We investigated whether patients with previous MTBI show this pattern of frontal activity reflected in more negative frontal alpha asymmetry (FAA) scores. Furthermore, in search for potential biomarkers of MTBI, we created a novel index, emotional modulation of FAA (eFAA) and investigated whether it correlates with subjective symptoms. EEG was recorded while subjects with previous MTBI and controls performed a computer-based reaction time task integrating different cognitive executive functions and containing either threat-related or emotionally neutral visual stimuli. Post-concussion symptoms and depression were assessed using the Rivermead Post-Concussion Symptoms Questionnaire (RPQ) and Beck’s depression inventory (BDI). Task-induced FAA was assessed and eFAA calculated by subtracting FAA in the context of neutral stimuli from FAA in the context of emotional stimuli. The MTBI group showed FAA scores reflecting relatively greater right-sided frontal activity compared to healthy controls. eFAA differentiated the symptomatic MTBI from non-symptomatic MTBI group and from healthy controls. eFAA also correlated with RPQ and BDI scores. In conclusion, FAA pattern previously linked with vulnerability to depression, was observed in patients with previous MTBI. Furthermore, eFAA has potential as a biomarker of altered affective brain functions in MTBI.

## Introduction

Mild traumatic brain injury (MTBI) is a common neurological injury with a worldwide incidence ranging from 100 to more than 600 per 100 000 people (Cassidy et al., 2004). While MTBI is usually associated with full recovery within 1 - 3 months (Belanger et al., 2005; McCrea et al., 2009), there is an estimated 10-20% minority who suffer from prolonged post-concussion symptoms (Ruff, 2005). Prolonged post-concussion symptoms include a variety of cognitive and emotional symptoms such as problems with concentration and memory, irritability, increased reactivity to emotional events, anxiety and depression. Despite several subjective symptoms that may significantly impair an individual’s daily functioning and quality of life, objective findings of brain injury or dysfunction are typically lacking, thus impeding access to appropriate rehabilitation and benefits. To that end, there is a need for objective biomarkers that reflect altered brain physiology underlying subjective symptoms after MTBI.

Prolonged post-concussion symptoms most likely reflect a multifactorial etiology (Ruff, 2005; Silverberg and Iverson, 2011), with psychosocial factors frequently emphasized in adverse outcomes. However, while biological processes are sometimes downplayed in the outcome of MTBI, they likely play an important role in recovery as well. Previous studies report changes in brain structure and function after MTBI, such as altered white matter integrity (Arfanakis et al., 2002; Kraus et al., 2007; Hartikainen et al., 2010b), connectivity (Lipton et al., 2009; Dean et al., 2015), cerebral perfusion (Barlow et al., 2017) and brain metabolism (Dean et al., 2015). There are also cellular level microscopic changes after MTBI that escape current clinical imaging techniques and are only visible in post-mortem studies. Post-mortem analysis of brains of four subjects dying within four months of acquiring MTBI showed multiple cell-level pathologies such as astrocytosis, axonal damage, perivascular injury and tau-protein accumulation (Tagge et al., 2018). At the level of higher brain functions, alterations in emotion-attention interaction (Mäki-Marttunen et al., 2015), working memory (Gosselin et al., 2011, 2012) and executive functions (Hartikainen et al., 2010b) have been reported after MTBI.

Electroencephalography (EEG) allows for investigating fine alterations in brain physiology underlying altered mental functions. Its millisecond temporal resolution corresponds to the timescale of rapid mental events. Event-related potentials (ERP) derived from EEG segments time-locked to specific cognitive processes and tasks allow investigation of cognitive and affective brain processes and their alterations due to neuromodulation, focal brain injury or MTBI (Hartikainen et al., 2012a; Mäki-Marttunen et al., 2015, 2017; Sun et al., 2015; Kuusinen et al., 2018). In addition to ERPs, different EEG frequency bands during cognitive or affective tasks may reflect alterations in thalamo-cortical activity due to neuromodulation (Sun et al., 2017a,b) or as a result of a brain disorder or head injury such as MTBI.

Frontal alpha asymmetry (FAA), i.e. asymmetry of alpha power between the left and right frontal region, has been linked with emotional processing (Davidson et al., 1990), emotional control (Sun et al., 2017a; Wang et al., 2018) and vulnerability to depression (Gotlib et al., 1998). However, in a recent review resting-state FAA was found to have a limited diagnostic value in depression partly due to the high degree of heterogeneity across studies (van der Vinne et al., 2017). Nevertheless, task-evoked FAA has been shown to reflect alterations in functioning of a key node of the limbic circuit, the anterior nucleus of the thalamus (ANT) (Sun et al., 2017a). Consequently, task-evoked FAA was suggested as a potential biomarker for the impact of neuromodulation on affective brain circuits (Sun et al., 2017a). FAA was also shown to be modulated by threat-related emotional stimuli and this emotional modulation was correlated with emotional modulation of reaction times (Sun et al., 2017a,b).

As alpha frequency is linked with idle or inhibited cortex (Klimesch et al., 2007; Händel et al., 2011), alpha power is thought to have an inverse relationship with cortical activity, with greater alpha power corresponding to less activity and vice versa. Therefore, relatively increased alpha power on the left frontal region in comparison to the right frontal region reflects decreased cortical activity on the left or increased activity on the right frontal region, or both (Coan and Allen, 2004). As FAA is a subtraction score where alpha power on the left is subtracted from alpha power on the right, lower or more negative score reflects relatively increased cortical activity on the right frontal region and/or relatively decreased cortical activity on the left frontal region. Lower or negative FAA values indicating relatively greater right frontal activity or left frontal hypoactivity have been associated with both current and previous episodes of depression (Henriques and Davidson, 1991; Gotlib et al., 1998; Thibodeau et al., 2006) as well as cognitive vulnerability to depression in never-depressed individuals (Nusslock et al., 2011). Cognitive vulnerability to depression along with left frontal hypoactivity on the other hand predicts future development of depression (Nusslock et al., 2011). As well as being linked to a predisposition to depressive behaviors or symptoms (Henriques and Davidson, 1991) FAA has been suggested as an objective marker of depression or vulnerability to it.

In addition to hemispheric lateralization of emotion, where the right hemisphere is thought to be predominant in emotion (Gainotti, 2019), especially negative emotion, and the left involved in positive emotion, there is hemispheric asymmetry in approach and withdrawal behaviors (Davidson, 1998). The left hemisphere is thought to be involved in approach behaviors while the right hemisphere involved in withdrawal behaviors. The approach-withdrawal model has been used as another theoretical framework for frontal alpha asymmetry, with asymmetry thought to reflect behavioral responses to emotionally valenced events (Coan and Allen, 2003; Harmon-Jones et al., 2010). Negative emotion and withdrawal behaviors are characteristic of depression and both are thought to lateralize to right hemisphere. Individuals who suffer from depression tend to withdraw from situations and stimuli, especially unpleasant ones (Davidson, 1998; Jesulola et al., 2015), whereas euthymic individuals express more approach-related behaviors (Davidson, 1998). Withdrawal-related behaviors and negative emotions are associated with increased activity in the right frontal region, while approach-related behaviors and positive emotions are linked with greater activity in the left frontal region with lower values and higher values of FAA correspondingly (Demaree et al., 2005).

We have previously used event-related potentials to detect brain injury–related changes in neural circuitries and dynamics (Kuusinen et al., 2018) and alterations in emotion-attention interaction due to MTBI (Mäki-Marttunen et al., 2015). In our study (Mäki-Marttunen et al., 2015), patients with MTBI allocated increased attentional resources to emotional stimuli as reflected in larger N2-P3 ERP peak-to-peak amplitude in the context of threat-related emotional stimuli compared to emotionally neutral stimuli. We hypothesized this excess attention allocation to threat might reflect impaired top-down control mechanisms that normally limit the impact of emotional stimuli on ongoing tasks. Excessive attention allocation to negative stimuli may eventually predispose individuals to emotional symptoms like depression. This effect, the so-called “negativity bias”, is seen especially in patients with depression whose attention is biased towards negatively-valenced stimuli (Gotlib et al., 2004), i.e. they may allocate more attentional resources to negative emotional stimuli for a prolonged period of time with difficulty in disengaging attention away from negative information (Gotlib and Joormann, 2010; Keller et al., 2019). Furthermore, depression is linked with impaired ability to control the impact of negative stimuli (Joormann, 2010) as well as problems with higher-level processing of emotion such as memory and interpretation (Foland-Ross and Gotlib, 2012).

Frontal alpha asymmetry has been used to study healthy populations and patients diagnosed with mood disorders, but it has not been frequently used to study neurological or neurosurgical patient populations like patients with MTBI who also exhibit alterations in affective brain processes, mood-related symptoms and vulnerability to depression. Our group has studied FAA in patients with refractory epilepsy treated with deep brain stimulation (DBS) (Sun et al., 2017a) and vagus nerve stimulation (VNS) (Sun et al., 2017b). These studies suggested that FAA could be used as a biomarker for alterations in functioning of affective brain circuits due to neuromodulation (Sun et al., 2017a,b). Turning on and off the anterior nucleus of the thalamus, a key node in the limbic circuitry, with high frequency electrical stimulation had a significant impact on FAA suggesting FAA reflects the functioning of limbic circuits and thus could be used as a biomarker not only for the impact of neuromodulation but also for the impact of brain disorder or injury on affective processes.

FAA seems to be a promising tool for objective assessment of alterations in affective brain functions due to MTBI possibly linked with emotion-related post-concussion complaints. It has been suggested that problems arising after brain trauma are associated with injury to connections between frontal and subcortical brain structures (McDonald et al., 2002; Hartikainen et al., 2010b; McAllister, 2011). Thus, it seems feasible that subtle damage to frontal-subcortical connections could influence FAA and expose individuals to depressive symptoms and behaviors. To expand our previous knowledge concerning alterations in emotion-attention interaction in patients with traumatic brain injury from ERP studies (Mäki-Marttunen et al., 2015, 2017; Kuusinen et al., 2018), we investigated whether there are alterations in FAA after MTBI. According to our knowledge, only one study by Moore and colleagues (Moore et al., 2016) has studied FAA in MTBI patients. They reported resting-state frontal alpha asymmetry indicative of greater activity in the right frontal region in patients with sports-related MTBI, albeit in completely symptom-free states at the time of testing (more than 9 months from the injury). Moreover, FAA correlated with self-reports of depressive/anxious symptoms, suggesting that alterations in FAA may reflect altered brain circuits associated with affective processes due to brain injury (Moore et al., 2016).

Alterations in the brain’s affective processes are most likely better reflected in FAA when measured during an emotionally engaging task (Coan et al., 2006; Allen and Reznik, 2015; Sun et al., 2017a,b). It is likely that for detecting alterations in affective circuits and functions they need to be engaged. Task-based recordings on the other hand require cognitive control and reduce mind wandering in contrast to resting-state recordings. In the current study, we used a behavioral task engaging several executive functions along with presentation of threat-related emotional stimuli. Threat-related emotional stimuli compete for the same attentional and cognitive control resources as the task and task-related stimuli. We compared FAA measures of healthy control subjects and subjects with previous MTBI. FAA was measured during a task which is reported to be more reliable than resting-state FAA measures in detecting differences in FAA on both individual- and group levels (Coan et al., 2006; Stewart et al., 2011, 2014).

We divided the MTBI group into two subgroups, those still reporting post-concussion symptoms and those being completely symptom free at the time of testing. We also created a novel emotional FAA index (eFAA) where FAA in the context of neutral stimuli was subtracted from that in the context of threat-related emotional stimuli, to isolate the sole impact of emotion on FAA. Our aim was to assess whether eFAA could serve as a biomarker to differentiate between symptomatic and non-symptomatic MTBI patients. Correlation analyses were conducted for eFAA and self-reports of depression and post-concussion symptoms. To our knowledge, this is the first study to assess task-related FAA and, specifically, the novel eFAA index in subjects with previous MTBI and whether it reflects the presence of post-concussion symptoms.

We hypothesized that patients with previous MTBI would have lower FAA values than healthy controls indicating relatively less alpha power on the right than left frontal region i.e. relatively greater right than left frontal cortical activity during the task in MTBI group. We also hypothesized that this effect would be pronounced in symptomatic MTBI patients differentiating them from non-symptomatic MTBI patients and that the eFAA index could be used as a biomarker to distinguish between symptomatic and non-symptomatic MTBI patients. We further hypothesized that removing depressed subjects from the analysis would reduce, but not remove, between-group differences. We postulated that eFAA would correlate with the amount of reported depressive symptoms and post-concussion symptoms.

## Materials and Methods

### Subjects

We studied 27 patients with previously acquired mild traumatic brain injury [15 males, 12 females, mean age 41.1 years (SD = 11.1 years, range 21-59 years), average time from injury to EEG recording 20.4 months (SD = 6.9 months, range 9-37 months), mean years of education 16.1 years]. Mild traumatic brain injury was classified according to the World Health Organization criteria for MTBI (Carroll et al., 2004). According to these criteria there is a biomechanical force to the head resulting in confusion, alteration of mental status, post-traumatic amnesia (PTA) or loss of consciousness (LOC) with PTA less than 24-h and LOC less than 30 min. Additionally, 30 min after the injury the Glasgow Coma Scale (GCS) should be at least 13. These patients were admitted to Tampere University Hospital emergency room between January 2010 and May 2012. Our control group consisted of 18 participants with no head injury who had been admitted to Tampere University Hospital emergency room due to an orthopedic injury, specifically ankle injury with no head injury [7 males, 11 females, mean age 40.7 years (SD = 12.1 years, range 23-60 years), mean years of education 15.9 years]. Event-related potential and behavioral data have been reported in our previous work showing that MTBI group allocated more attention to threat-related stimuli than the control group (Mäki-Marttunen et al., 2015).

Based on Rivermead Post Concussion Symptoms Questionnaire (RPQ) (King et al., 1995) the MTBI group was divided into those reporting any post-concussion symptoms (MTBI Symptomatic, *n* = 15) and those reporting no post-concussion symptoms (MTBI Non-symptomatic, *n* = 12) at the time of testing. Exclusion criteria for both groups were substance abuse and neurological or psychiatric conditions diagnosed prior to the injury, previous MTBI in orthopedic patients and history of more than one MTBI in the MTBI group, previous neurosurgical procedures and markedly impaired vision/hearing. A written informed consent was provided by all participants and the study followed Declaration of Helsinki guidelines for use of human subjects. The Ethical Committee of Tampere University Hospital provided their approval for the study.

### The Executive Reaction Time Test

**FIGURE 1:**
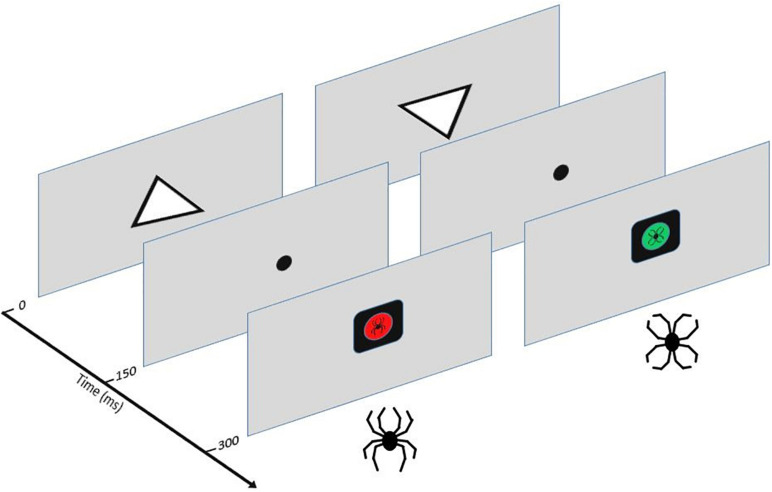
The Executive RT Test (Hartikainen et al., 2010b). Participants were seated in front of a computer screen at one meter in a sound-attenuated room. Subjects were required to respond as fast and accurately as possible to the orientation of a triangle using their middle or index finger in a Go-trial indicating the triangle was pointing up or it was pointing down, respectively. In a NoGo-trial, subjects were supposed to withhold from responding. A trial started with an upright or inverted white triangle presented in the middle of the screen for 150 ms. After the onset of the triangle, a fixation dot appeared for 150 ms. This was followed by a black square with a colored circle and an emotional figure inside it. The colored circle and the emotional figure were response cues. There were two possible response sets: the participants were either asked to pay attention to the color (red or green) and ignore the emotional figures (flower or spider) as task-irrelevant distractors or pay attention to the emotional figures and ignore the colors. The stimuli (color or emotional figure) attended to indicated the rule for responding, e.g., when color was attended to the red-color indicated a Go-trial and the green-color a Nogo-trial or vice versa. When the emotional figure was attended to the spider figure indicated a Go-trial and the flower a Nogo-trial or vice versa. In case of a Go-trial, participants indicated the orientation of the previously presented triangle by pressing a button on a response pad. The emotional stimuli were black-line drawings of a spider (threat-related emotional stimulus) and a flower (emotionally neutral stimulus) constructed the exact same line elements as the spider but in a different configuration. This allowed control for the impact of low-level properties of visual stimuli, like color, contrast, and complexity.

The Executive Reaction Time test (The Executive RT test) is a computer-based Go/NoGo test integrating assessment of different executive functions and impact of threat-related stimuli on cognitive performance and brain responses (Erkkilä et al., 2018; Hartikainen et al., 2010b). In this study a slightly modified version of the Executive RT test was used, where the threat-related stimuli were not only task-irrelevant distractors but also task-relevant stimuli depending on the rule for responding (Mäki-Marttunen et al., 2015). Task performance and dealing with potential threat are thought to compete for the same attentional and cognitive control resources and this resource competition and the extent of it is reflected in both electrophysiological data and performance (Kuusinen et al., 2018; Mäki-Marttunen et al., 2015). The test requires the engagement of different executive functions (working memory, selective attention, response inhibition, shifting and emotional control) and has previously been shown to detect mild executive dysfunction in MTBI patients with post-concussion symptoms (Hartikainen et al., 2010b). A schematic picture of the Executive RT test and task description are presented in Figure 1.

Each block of the task consisted of 64 trials and the same response rule continued throughout the whole block. When subjects completed one block of trials the response rule changed. Altogether, 16 blocks were completed in the study, resulting in each response rule repeated four times. All in all, participants had to hold the orientation of the triangle and the current response rule in their working memory. They had to flexibly shift between changing task rules, maintain their attention at task-relevant stimuli and ignore task-irrelevant stimuli, react to the Go response cue and withhold their response when seeing the NoGo cue according to the current rule.

### EEG Recording and Processing

We used 64 Ag/AgCl active electrodes and the QuickAmp Amplifier (Gilching, Germany) to record EEG. Impedance was kept below 5 kOhms and the sampling rate used was 500 Hz. After recording, EEG analysis was performed offline using BrainVision Analyzer 2 software (Brain Products, GmbH, Germany). To improve processing speed, sampling rate of the data was reduced to 250 Hz. Data was first filtered to 0.01–70 Hz and ocular movements removed with semi-automatic independent component analysis–based ocular correction algorithm. Data was then re-referenced to the Cz electrode and further filtered to 0.1-30 Hz using bandpass filters. Data was segmented according to the emotional stimuli presented (emotional, neutral), relevance of the emotional stimuli to the task (relevant, irrelevant) and Go- vs. NoGo condition. Segments created were two seconds long starting at the presentation of the triangle at 0 ms and baseline corrected to the first 200 ms of the segment. Another artifact removal was conducted excluding amplitudes larger than 80 μV and smaller than −80 μV. Alpha power (μV^2^/Hz) for the segments was calculated using the Fast Fourier Transformation and thereafter averaged for each subject, electrode and condition. Thus, task-induced eyes-open alpha power was obtained during the active task for each different trial condition.

Alpha power in the defined alpha frequency of 8-12 Hz was exported for statistical analysis as area under the curve. We used the F3 and F4 electrodes for calculating FAA: first, alpha power in these electrodes was log-transformed and then the values in F3 were subtracted from values in F4 (FAA = ln(F4) – ln(F3)).

### Questionnaires

To assess post-concussive, depressive and executive function-related symptoms, questionnaire data was acquired from both the MTBI and the Control groups. The RPQ (King et al., 1995) is a self-report questionnaire listing common post-concussion related symptoms. Results of this questionnaire were used to define whether participants were symptomatic or not at the time of testing and symptom scores were also used in correlation analysis. We also separated symptoms listed in the questionnaire that were associated with alterations in emotional processing (irritability, depression or tendency to cry, lack of patience, restlessness) creating a separate category “RPQ Emotional” and used the scores in this category in correlation analysis. Both MTBI and control groups also completed Becks Depression Inventory (Beck et al., 1996) (BDI) to assess whether they experienced depressive symptoms.

Behavior Rating Inventory for Executive Functions – Adult version (BRIEF-A) questionnaire is a standardized measure of executive functions and self-regulation in daily life based on self-report (Roth et al., 2005). There are different categories in the BRIEF-A questionnaire that reflect different aspects of executive functions. In this study only the Global Executive Composite (GEC), i.e. the total score of the questionnaire and the Emotional Control category assessing challenges in emotional control were used for further analysis.

### Statistical Analysis

Statistical analysis was conducted with R version 3.6.1 (R Core Team, 2015). Normal distribution of the data was assessed with histograms and Q-Q plots and checking for normality of the residuals (for data analyzed with ANOVA). FAA data was strongly skewed. Applying the natural logarithm when calculating FAA (see above) reduced this to some extent, however, not totally correcting for left-skewed distribution of the data. Finally, data was normalized removing two outliers that clearly created the left skew in the data based on average FAA values of the groups. Outliers were defined as ± 2.5 x SD + the mean. In the current data outlier values were ± 2.5 x 0.41 −0.14, i.e. below −1.165 and above 0.885. One subject from the MTBI group and one from the Control group were left out from the analysis, leaving 26 patients and 17 control subjects in the final sample. Equal variances were confirmed with Levene’s test using package car (Fox and Weisberg, 2019). Psych (Revelle, 2019) and ggplot2 (Wickham, 2016) packages were used for describing and visualizing the data.

FAA data was analyzed using mixed model ANOVA (repeated and between group measures) with the R ez package (Lawrence, 2016). We used Group (MTBI, Control) as a between-subjects factor and Emotion (Emotional, Neutral) and Relevance (Relevant, Irrelevant) as within-subjects factors. Significant interactions were further analyzed with post-hoc ANOVAs or independent-sample t-tests. We used the Holm correction for multiple comparisons. As the FAA data based on analysis of the residuals was still not completely normal after transformations and outlier removal, we confirmed the significant results with non-parametric tests on the original data (with the outliers not excluded) using Wilcoxon Rank Sum test and Kruskal-Wallis test. Similar analysis of variance on the FAA data was also run to compare alpha asymmetry between three groups. In this analysis Group factor had three levels (MTBI Symptomatic, MTBI Non-symptomatic, Control) instead of two (MTBI, Control).

We also did an analysis where we excluded patients who reported depressive symptoms in the BDI questionnaire (10 or more points in the BDI corresponding mild clinical depression), as depression could affect the results of alpha asymmetry analysis. There were 4 subjects in the MTBI group and 1 in the Control group with 10 or more points, thus they were removed from this analysis.

To see whether alpha asymmetry in the context of threatening stimuli could serve as a biomarker distinguishing between symptomatic and non-symptomatic MTBI patients and healthy controls, we created a novel emotional FAA index (eFAA). This was done by calculating a subtraction score that isolated the sole impact of threat on FAA by removing FAA value in context of emotionally neutral stimuli from FAA value in context of threat-related emotional stimuli. This subtraction score was calculated for each patient, and it was used to run another ANOVA with Group (MTBI Symptomatic, MTBI Non-symptomatic, Control) as a between-subjects factor and Relevance (Relevant, Irrelevant) as a within-subjects factor. One subject from the control group was removed from this analysis as an outlier based on above described criteria.

To evaluate whether self-reports of depressive symptoms and post-concussion symptoms are associated with the novel index reflecting emotional modulation of FAA (eFAA), we correlated eFAA with the selected summary questionnaire scores (BDI, RPQ, RPQ Emotional, BRIEF GEC, BRIEF Emotional Control). In addition, we correlated eFAA with age, time post-injury (from injury to the EEG recording) and PTA to assess the influence of age and head injury related factors. While all of the subjects were classified as mild TBI, PTA is a clinical measure thought to reflect the severity of the injury at the acute phase. Analysis was conducted for all three groups (MTBI Symptomatic, MTBI Non-symptomatic, Controls) together, except for correlation between eFAA and time-post injury and PTA, as this data was only available for the MTBI group. As the questionnaire data was strongly skewed, we used Spearman’s correlation. One subject in eFAA-BDI correlation and one in eFAA-PTA correlation were removed as outliers. Other analysis of the questionnaire data is reported more thoroughly in our previous work (Mäki-Marttunen et al., 2015).

## Results

### Frontal Alpha Asymmetry (FAA)

**FIGURE 2:**
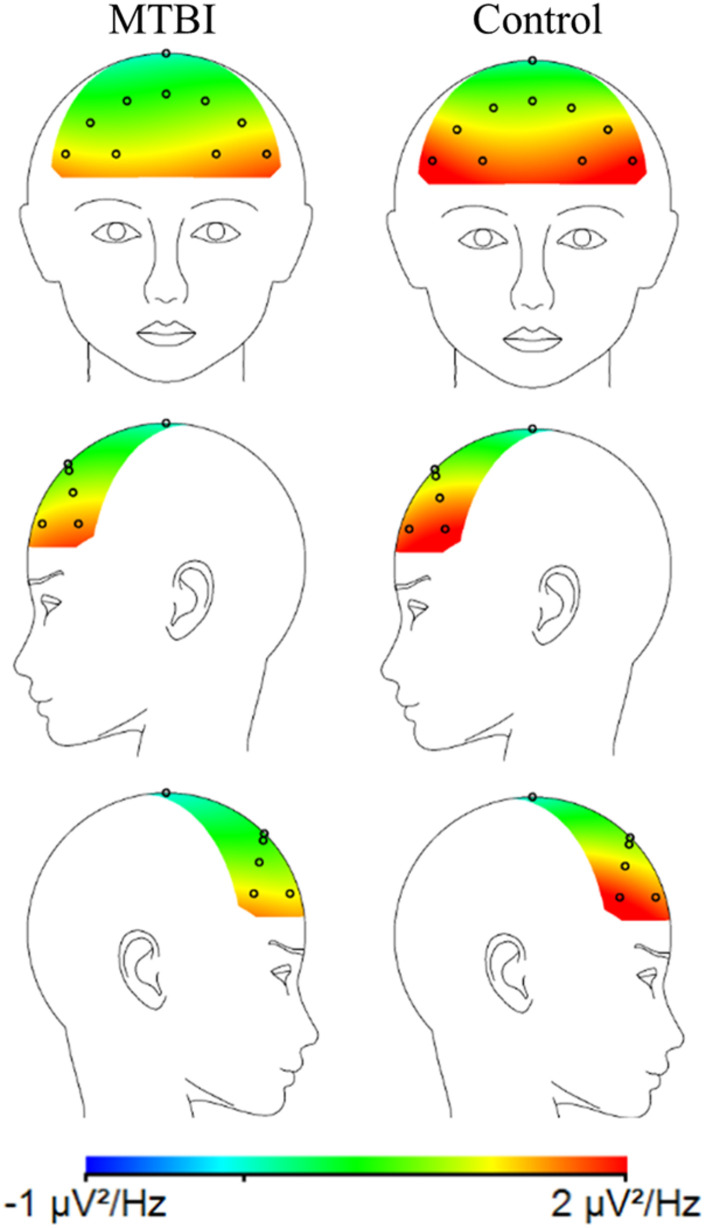
A topographic plot illustrating the average alpha power (μV^2^/Hz) for the MTBI group (on the left) and the Control group (on the right) in the frontal electrodes. The MTBI group had relatively more negative FAA (lnF4-lnF3) than the Control group, i.e., relatively less alpha power on the right (F4) than left frontal (F3) electrodes. This is thought to indicate greater cortical activity on the right compared to the left frontal region.

When MTBI and Control groups were compared, the MTBI group showed more negative FAA scores than the Control group, indicative of relatively less alpha power on the right than left frontal region in MTBI (Figure 2) reflecting greater right than left frontal cortical activity (Main effect of Group, *F*(1, 41) = 6.36, *p* = 0.016, η^2^_G_ = 0.13; MTBI vs. Controls, −0.17 ± 0.32 vs. 0.06 ± 0.25), see Figure 2 and Table 1. This difference in FAA remained significant after removing subjects reporting more than 10 points in the BDI questionnaire (MTBI = 4 subjects, Control = 1 subject, Main effect of Group, *F*(1, 36) = 4.78, *p* = 0.035, η^2^_G_ = 0.11; MTBI vs. Controls, −0.12 ± 0.28 vs. 0.07 ± 0.25). No other significant main effects or interactions were detected when analyzing data between two groups.

**TABLE 1:** Average FAA, eFAA and alpha power (μV^2^/Hz) at F3 and F4 electrodes, the corresponding logarithms (ln) and standard deviation (SD) for each group and condition (neutral, threat). FAA differentiated MTBI group from the Control Group while eFAA differentiated the Symptomatic MTBI group from both the Non-symptomatic MTBI group and the Control Group. Non-Symp = Non-symptomatic MTBI group, Symp = Symptomatic MTBI group.

**Group**	**FAA**	**SD**											
**Control**	0.058	0.252											
**MTBI**	−0.172	0.318											

**Group**	**eFAA**	**SD**	**Emotion**	**F4**	**SD**	**F3**	**SD**	**ln(F4)**	**SD**	**ln(F3)**	**SD**	**FAA**	**SD**

**Control**	0.003	0.032	**Neutral**	5.004	9.588	5.225	10.630	0.788	1.120	0.732	1.195	0.057	0.251
			**Threat**	5.000	9.399	5.232	10.525	0.798	1.121	0.738	1.200	0.060	0.255
**Non-Symp**	0.021	0.034	**Neutral**	4.749	4.200	5.773	4.886	1.180	0.906	1.395	0.872	-0.215	0.336
			**Threat**	4.813	4.320	5.760	4.971	1.189	0.915	1.383	0.885	-0.194	0.355
**Symp**	−0.019	0.036	**Neutral**	2.435	2.421	3.026	3.486	0.549	0.777	0.684	0.845	-0.135	0.291
			**Threat**	2.467	2.503	3.088	3.524	0.545	0.799	0.699	0.860	-0.154	0.299

When the MTBI group was divided into Symptomatic and Non-symptomatic groups and the analysis was conducted as a three-group comparison (MTBI Symptomatic, MTBI Non-symptomatic, Control), Main effect of Group was detected (*F*(2, 40) = 3.26, *p* = 0.049, η^2^_G_ = 0.14), however, post-hoc analysis remained nonsignificant. Difference between Non-symptomatic MTBI group and Controls was approaching significance (*p* = 0.07). Non-symptomatic and Symptomatic MTBI did not differ (p = 0.61). An interaction effect of Group × Emotion was also detected (*F*(2, 40) = 4.46, *p* = 0.018, η^2^_G_ = 0.0007). Data was divided by Emotion and groups were compared in Neutral and Emotional conditions. In Neutral condition, Main effect of Group reached significance (*F*(2, 40) = 3.41, *p* = 0.043, η^2^_G_ = 0.15) but post-hoc test did not result in significant results. In the Emotional condition, Main effect of Group was close to reaching significance (*F*(2, 40) = 3.12, *p* = 0.055, η^2^_G_ = 0.13).

### Emotional Modulation of Frontal Alpha Asymmetry (eFAA Index)

**FIGURE 3:**
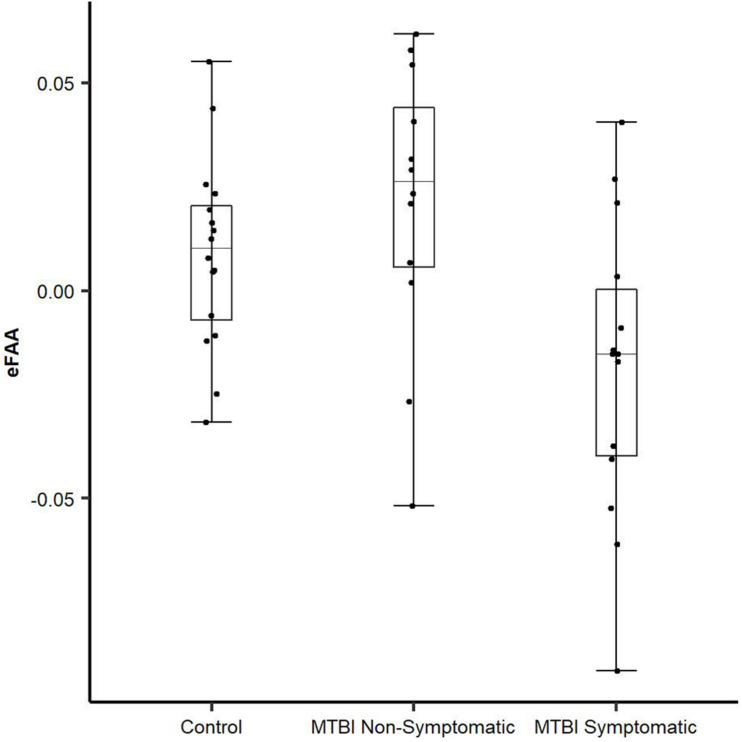
Novel eFAA index differentiates MTBI subjects with symptoms from those without. eFAA, a subtraction score isolating the impact of threat on FAA differed significantly between Symptomatic MTBI and Non-symptomatic MTBI groups and between Symptomatic MTBI group and healthy controls. Symptomatic MTBI group had more negative eFAA index compared to Non-symptomatic MTBI group and healthy controls, indicating altered modulation of FAA by threat in symptomatic MTBI group.

To be able to extract the sole impact of threat-related stimulus on FAA and to assess whether alpha asymmetry in the context of threat has potential as an objective biomarker of brain dysfunction reflecting post-concussion symptoms, we conducted an additional ANOVA using the eFAA index, where FAA in context of emotionally neutral stimuli was subtracted from FAA in context of threat-related stimuli. There was a Main effect of Group (*F*(2, 39) = 5.69, *p* = 0.007, η^2^_G_ = 0.23) and when groups were compared, eFAA in the Symptomatic MTBI group differed significantly from both eFAA in the Non-Symptomatic MTBI group (*t* = 2.87, df = 23.717, *p* = 0.009; Symptomatic vs. Non-symptomatic −0.019 ± 0.04 vs. 0.02 ± 0.03) and the Control group (*t* = 2.46, df = 21.562, *p* = 0.022; Control Group 0.003 ± 0.03), see Figure 3 and Table 1. No other significant main effects or interactions were detected. *Post-hoc p*-values were corrected for multiple comparison using the Holm method.

### Questionnaires and Correlation Analysis

To investigate whether the novel index eFAA correlates with self-reports of depressive symptoms, post-concussion symptoms and executive function problems as well as brain injury related factors, we ran a Spearman’s correlation analysis of the data. All three groups were included in the analysis (MTBI Symptomatic, MTBI Non-symptomatic, Control) except for the correlation for eFAA and time post-injury and PTA, as this data was only available for the MTBI group.

**FIGURE 4:**
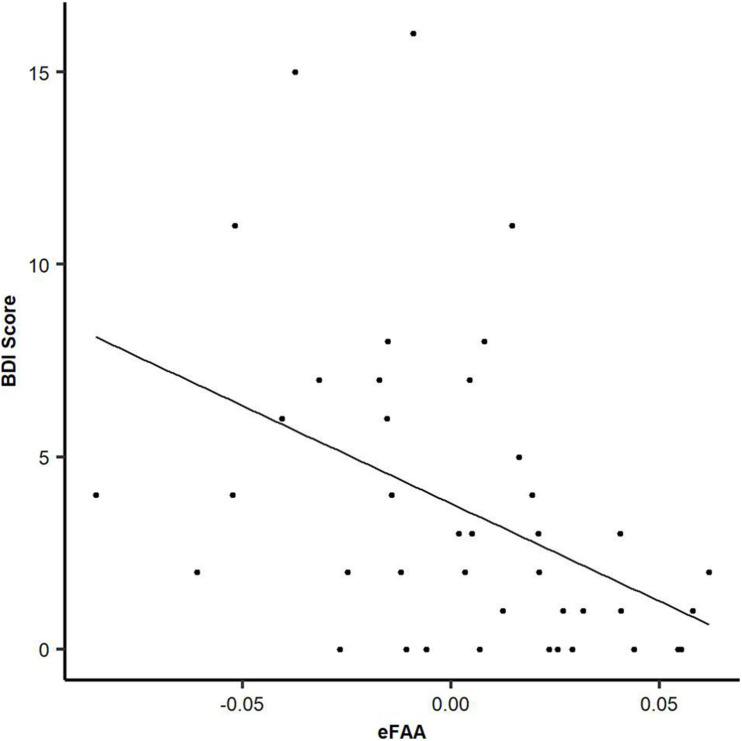
Objective eFAA index correlates with subjective depression symptoms. There was a significant negative correlation between BDI score and eFAA (threat modulation of FAA). When BDI score was higher, eFAA was more negative. All three groups (MTBI Symptomatic, MTBI Non-symptomatic, Control) are included in the analysis and in the scatter plot.

There was a significant negative correlation between eFAA index and BDI results (ρ = −0.52, *p* < 0.001), showing that subjects with higher score in BDI questionnaire had more negative eFAA index, see Figure 4. More negative eFAA index reflects smaller FAA in context of threat-related stimuli than neutral stimuli. There was also a significant negative correlation between eFAA index and currently reported post-concussion symptoms (RPQ) (ρ = −0.34, *p* = 0.025), with subjects reporting more post-concussion symptoms having more negative eFAA index. There was a tendency for negative correlation between emotional control category in the BRIEF-A questionnaire and eFAA index (ρ = −0.26, *p* = 0.089). There was also a positive correlation between eFAA and PTA, showing that MTBI patients with longer PTA had more positive eFAA index (ρ = 0.41, *p* = 0.044). BRIEF general score, emotion-related post-concussion symptoms, age and time post-injury did not significantly correlate with eFAA index.

## Discussion

According to our hypothesis, we detected more negative FAA indicative of greater right frontal activity in the MTBI group compared to the control group. Subjects with previous MTBI had FAA pattern typically seen in individuals vulnerable to depression (Henriques and Davidson, 1991; Gotlib et al., 1998; Thibodeau et al., 2006; Nusslock et al., 2011). While the overall FAA did not distinguish symptomatic and non-symptomatic MTBI groups, the novel index eFAA did. eFAA reflects modulation of FAA by threat, with the emotionally neutral but otherwise identical condition subtracted, thus isolating the sole impact of threat on FAA. eFAA correlated negatively with both current post-concussion symptoms and BDI scores, linking this objectively derived brain physiology-based index with subjectively experienced symptoms. eFAA showed potential as an objective biomarker of brain dysfunction reflecting post-concussion symptoms as well as depression symptoms. In addition to providing a biomarker for the impact of brain injury on affective brain functions, eFAA suggests an organic basis for the subjectively experienced symptoms. eFAA may be used in future studies to further illuminate neural mechanisms underlying susceptibility to prolonged post-concussion as well as mood-related symptoms after MTBI. FAA without engagement of affective brain circuits seems to lack the sensitivity to differentiate subjects with symptomatic and non-symptomatic MTBI and the ability to reflect the subjective post-concussion or depression symptoms.

To detect alterations in affective circuits responsible for emotion-related behaviors, symptoms and their control, it may be essential to assess brain functions in the context of fear-related stimuli such as the biologically relevant threat-related spider stimuli used in the current study. Threat-related stimuli have prioritized access to attention networks (Vuilleumier and Schwartz, 2001) and tend to compete for attentional and cognitive control resources with other task-related stimuli and processes (Hartikainen et al., 2000, 2007, 2010a, 2012b). Alterations in the brain’s affective functions may remain uncovered when brain physiology is measured in emotionally neutral conditions such as is the case in most studies using resting state or task-induced FAA. The difference in FAA between symptomatic and non-symptomatic patients was detected only with the novel index that isolated the impact of threat-related stimuli on FAA, suggesting there might be differences in fear-related emotional processing between subjects that have fully recovered from MTBI and subjects who remain having some post-concussion symptoms. While the MTBI group in general differed from the control group in the overall FAA, the eFAA differentiated MTBI subjects with symptoms from those without symptoms. To our knowledge, no previous study has used task-induced, threat-related FAA to differentiate non-symptomatic and symptomatic MTBI groups. In addition to controlling for the impact of physical properties of the visual stimuli, the novel eFAA index controls for the individual variations in baseline FAA.

Subjects reporting more post-concussion symptoms and subjects with higher BDI-score at the time of testing had a more negative eFAA index. eFAA was negatively correlated with self-reported scores assessed by the BDI questionnaire and the current amount of reported post-concussion symptoms assessed by the RPQ. The BDI is used for screening of depression while the RPQ (King et al., 1995) screens for cognitive, emotional and somatic post-concussion symptoms, with higher score in the BDI reflecting more depression symptoms or more severe depression and more post-concussion symptoms in the RPQ, respectively. While a subcategory of emotion-related post-concussion symptoms did not correlate with eFAA there was a slight tendency for negative correlation between emotional control category in the BRIEF-A questionnaire and eFAA index.

Depressive symptoms are thought to be associated with post-concussion symptoms (Yrondi et al., 2017) and risk of major depression after MTBI is almost two times higher compared to risk after non-head orthopedic trauma (Stein et al., 2019). While post-concussion symptoms are sometimes seen in non-head trauma populations as well, the symptom pattern differs from that of MTBI, which correlates highly with depression and anxiety (de Koning et al., 2016). Pre-injury anxiety and depression are linked with greater post-concussion symptom severity, however controlling for pre-injury symptoms, the change in symptoms due to MTBI did not differ between those with pre-injury anxiety and depression and those without in a study assessing post-concussion symptom reporting (Karr et al., 2020). Interestingly, we found a positive correlation between eFAA and PTA, showing that patients with longer PTA had more positive eFAA, in contrast to negative correlation between eFAA and RPQ and BDI scores. Longer PTA does not predict depression or post-concussion syndrome (PCS) and it still remains a challenge to predict outcome from any acute phase clinical measures including PTA in MTBI. On the contrary, long PTA or having no memory of the traumatic event might even protect one from intrusive memories at the acute stage (Bryant et al., 2009) and the development of PCS (Gil et al., 2005). Furthermore, with lack of objective evidence for brain injury or dysfunction in uncomplicated MTBI it is difficult to predict functional recovery and impairment. As depression and anxiety after MTBI have been shown to be important precursors of functional impairment, it is essential to assess mental health and emotion-related symptoms after MTBI (Zahniser et al., 2019). To that end, the novel biomarker of MTBI, eFAA, based on emotion-related brain functions, i.e. the difference in brain response to emotionally threatening and neutral stimuli, correlates with both measures reflecting the acute severity of the injury as well as the outcome measures including mood and subjective post-concussion symptoms.

Threat or fear-related stimuli or events are frequently used to study anxious or depressed populations to assess alterations in fear-processing. Anxiety and depression are intricately intertwined as post-concussion symptoms. Rodents with MTBI show fear- and anxiety-related behaviors, increased fear-learning and generalization of fear when compared to control animals, along with molecular changes in regions involved in fear-related behaviors such as the amygdala and in regions involved in emotional control, such as the prefrontal cortex (Statz et al., 2019). These behavioral and molecular changes after MTBI linked with fear and anxiety observed in animal studies suggest biological factors contribute to anxiety after MTBI. Further evidence comes from human studies reporting increased anxiety and depression after MTBI as well as evidence of structural changes contributing to these symptoms, e.g. a thinning of the right orbitofrontal cortex involved in downplaying negative emotions (Epstein et al., 2016). Focal injury to OFC is a frequent consequence of traumatic brain injury (TBI) and known to be involved in dysregulation of attention to emotion, possibly contributing to the challenges in emotional and social behaviors experienced by patients with OFC injury (Hartikainen and Knight, 2003; Hartikainen et al., 2012a; Kuusinen et al., 2018; Mäki-Marttunen et al., 2017). The internal structural configuration of the skull, such as surrounding sharp bony ridges of the skull which hold the brain in place, make the orbitofrontal cortex especially vulnerable to head injury. Because of this, it may well be that this area is involved in some of the subjectively experienced emotional symptoms in MTBI.

While the current study does not address the specific neural mechanisms contributing to altered FAA nor to the altered emotional modulation of FAA (eFAA) after MTBI, we can infer that MTBI results in altered activity of fronto-thalamic networks, distinctly and dynamically affecting the left and right hemispheres leading to alterations in typical emotion regulation. This was seen in eFAA correlating negatively with BDI and current post concussion symptoms and positively with PTA. Thus, eFAA can be thought of as a biomarker of MTBI reflecting both acute severity of the injury as measured with PTA as well as long-term outcomes as measured by BDI and RPQ. Previous studies have reported alterations in frontal white matter integrity (Strain et al., 2013) and generally reduced functional connectivity of emotion processing areas (McCuddy et al., 2018) in patients with depressive symptoms after MTBI. Altered balance between right and left frontal cortical activity may reflect alterations in the brain’s affective and emotional control functions. For example, decreased left frontal activity is linked with more emotion regulation difficulties (Zhang et al., 2020). Such alterations may be one of the underlying mechanisms of depression symptoms and vulnerability to depression after MTBI. Our results showing task-induced FAA indicative of greater right frontal activity in subjects with previous MTBI resemble those of Moore and colleagues who obtained similar results in a resting-state experiment (Moore et al., 2016). Depressed subjects with previous MTBI show altered functional connectivity (Banks et al., 2016; McCuddy et al., 2018) similar to that seen in depressed subjects without previous MTBI (Alhilali et al., 2015; Chen et al., 2008). While only a few of our subjects had BDI scores indicating mild depression and none had major depressive disorder, it may well be the altered FAA observed in the current study in subjects with previous MTBI reflect similar changes in functional connectivity as previously reported due to depression.

In comparison to healthy controls, FAA indicative of greater right frontal activity in the current study was seen in the MTBI group, including patients who did not complain of any post-concussion symptoms. We speculate that organic dysfunction could contribute to altered FAA after MTBI with psychosocial and cognitive factors likely contributing to whether this brain injury-based vulnerability develops into post-concussion symptoms or clinical depression. It seems that both symptomatic and non-symptomatic MTBI groups have the same underlying brain physiology-based vulnerability reflected in FAA, but the MTBI group without symptoms is more efficient in rapid emotional control in the face of unexpected threat. It may well be that it is a matter of efficiency of the rapid emotional control circuits reflected in eFAA index that distinguish MTBI without symptoms from those with prolonged symptoms.

We have previously suggested that FAA could be used as a potential biomarker for the effect of neuromodulation on brain’s affective circuits (Sun et al., 2017a). We showed FAA reflected the functioning of a key node of the limbic circuit, i.e. anterior nucleus of the thalamus (ANT). With high-frequency electric stimulation used in deep brain stimulation (DBS) we were able to basically turn on and off ANT and observe online the impact of ANT on FAA. DBS at ANT resulted in altered FAA indicative of greater right frontal activation along with greater impact of threat on reaction times (RTs). Thus, alteration in FAA was linked with alteration in emotion modulated behavior. In that study we also calculated a subtraction score similar to eFAA that isolated the impact of threat-related stimuli on FAA and found this index to correlate with a subtraction score obtained in a similar manner to isolate the impact of threat on RTs. To that end, while we did not call it eFAA yet, nevertheless this index was linked with emotion modulated behavior. DBS at ANT is used to treat refractory epilepsy and while it is an effective treatment, depression symptoms have been mentioned as a side effect (Fisher et al., 2010). Another treatment of refractory epilepsy and depression, vagus nerve stimulation (VNS), resulted in FAA values indicative of greater right frontal activation as well (Sun et al., 2017b). It is of interest we found a similar pattern of FAA in the current study of MTBI patients as observed in patients with refractory epilepsy undergoing ANT-DBS and VNS in contrast to conditions when these neuromodulation treatments were turned off.

What links these very different clinical populations is alterations in activity of fronto-thalamic cognitive and emotional control circuits (Hartikainen et al., 2010b, 2014). Thus, the potential of task-induced FAA in threatening context or eFAA as a biomarker is not limited to MTBI or neuromodulation, but rather encompasses all clinical populations that have altered functioning of fronto-thalamic control circuits. Such clinical populations include neurodevelopmental disorders such as attention deficit/hyperactivity disorder (ADHD) with many of the cognitive and affective symptoms overlapping with those of MTBI. This is evident from the BRIEF questionnaire, that captures many of the typical symptoms of TBI even though it was originally designed to assess executive dysfunction in ADHD. A link between ADHD symptoms and alpha asymmetry has been previously reported by Keune et al. (2015) suggesting altered asymmetry of frontal circuits and further pointing to potential in biomarkers based on measurement of this asymmetric activation in this clinical population as well (Keune et al., 2015). While future studies are needed before final conclusions of clinical applicability of eFAA can be made the prospects of an objective biomarker of alterations of emotional brain processes are vast and emphasized by the current lack thereof.

Intact fronto-thalamic cognitive control circuits are essential for efficient emotion regulation as some of the resources used for both cognitive and emotional control are shared (Ochsner and Gross, 2005; Langner et al., 2018). Adaptive emotion regulation strategies rely heavily on cognitive control, also called executive functions, and impaired executive functions increase the risk of depression (Letkiewicz et al., 2014). Fronto-thalamic networks responsible for executive functions are vulnerable to injury and dysfunction due to head trauma (Hartikainen et al., 2010b). Dysfunction of these networks result in impaired cognitive (Peräkylä et al., 2017) and/or emotional control (Hartikainen et al., 2014; Sun et al., 2015), making patients susceptible to depression symptoms (Fisher et al., 2010). We have previously shown that subjects with persistent symptoms after MTBI have compromised executive functions potentially linked with alterations in integrity of fronto-subcortical circuits (Hartikainen et al., 2010b) and that subjects with previous MTBI allocate more attention to threat-related emotional stimuli than control subjects (Mäki-Marttunen et al., 2015). Prolonged attention to negative emotional stimuli is frequently seen in depression and in a well-known phenomenon called negativity bias (Gotlib and Joormann, 2010; Keller et al., 2019). Such negativity bias after MTBI may reflect dysfunction in frontal control systems leading to similar phenomena that are observed in depressed subjects, such as an inability to inhibit attention to negative emotional stimuli or limit their impact, thus predisposing one to depressive symptoms (Joormann and Gotlib, 2008; Koster et al., 2011; Bistricky et al., 2014). We suggest this may be one of the mechanisms contributing to the high prevalence of depression after MTBI.

Despite several strengths, there are some limitations to our study. Even though we found a difference in threat-related eFAA between symptomatic and non-symptomatic MTBI groups and between symptomatic MTBI group and healthy controls, the clinical applicability of these results remains to be confirmed in future studies. As the groups, and especially the subgroups of MTBI patients were small, the results should be considered as preliminary and conclusions made with caution. None of the patients were diagnosed with PCS, but rather they reported to have some ongoing post-concussion symptoms as measured by the RPQ. Further studies with patients diagnosed with PCS using FAA are needed to validate the usefulness of FAA in this clinical population. Neither were our subjects diagnosed with clinical depression, but rather there was an association with eFAA and depression-related symptoms. This is in line with lower FAA scores indicative of greater right frontal activity reflecting vulnerability to depression (Henriques and Davidson, 1991; Nusslock et al., 2011). Furthermore, it may well be that FAA evoked in threat-related context could be even more tightly linked with anxiety than depression as anxiety is thought to be more directly linked with altered fear processing than depression which is a common concomitant of anxiety and intertwined with it. It may be that both depression-related symptoms, as well as other post-concussion symptoms, are more prevalent in those subjects who suffer from anxiety. To that end, in future studies we recommend using anxiety inventories along with depression and other post-concussion symptoms inventories to address the role of anxiety and its correlation with FAA and threat-modulation of FAA. Finally, before task-induced FAA and eFAA could be utilized as clinical biomarkers, more research on different factors impacting these indices is required. Such factors that could impact FAA are, for example, hormonal status, gender, age, developmental disorders, medications and other conditions impacting affective brain functions.

In conclusion, we found MTBI to be associated with lower FAA scores indicative of greater right frontal activity independent of symptom status. Moreover, the current study presented a novel brain activity based biomarker of emotional control circuits. Emotional modulation of FAA, eFAA, distinguished symptomatic MTBI patients from non-symptomatic ones and reflected subjective post-concussion and depression symptoms after MTBI. eFAA depicts altered neural activity in response to emotional events that likely underlie subjective symptoms and vulnerability to depression after MTBI. To that end, eFAA has potential as a biomarker of altered affective brain functions with applicability in both clinical and research fields.

## Data Availability Statement

Restrictions apply to the datasets. The datasets for this manuscript are not publicly available because of hospital policies and signed consents by the participants not covering public distribution of data. Requests to access the datasets should be directed to KH (kaisa.hartikainen@live.com).

## Ethics Statement

The studies involving human participants were reviewed and approved by the Ethical Committee of Tampere University Hospital. The participants provided their written informed consent to participate in this study.

## Author Contributions

VK had the main responsibility for collecting, processing and analyzing the data, and writing the article. JP contributed to data analysis and writing the article. LS contributed to data collection, data analysis, and writing the article. KO and KH contributed to interpreting the results and writing the article. KH designed the study, invented the eFAA index, supervised the project and had main responsibility for the scientific, and writing process. All authors contributed to the revision process, read and approved the final version of the article.

## Conflict of Interest

The authors declare that the research was conducted in the absence of any commercial or financial relationships that could be construed as a potential conflict of interest.
